# Aerial Surveys Give New Estimates for Orangutans in Sabah, Malaysia

**DOI:** 10.1371/journal.pbio.0030003

**Published:** 2004-12-07

**Authors:** Marc Ancrenaz, Olivier Gimenez, Laurentius Ambu, Karine Ancrenaz, Patrick Andau, Benoît Goossens, John Payne, Azri Sawang, Augustine Tuuga, Isabelle Lackman-Ancrenaz

**Affiliations:** **1**Kinabatangan Orang-utan Conservation Project, SandakanSabahMalaysia; **2**CEFE/CNRS, équipe Biométrie et Biologie des populationsMontpellierFrance; **3**Institut de l'Ingénierie de l'Information de Santé, équipe TIMBFaculté de Médecine, La Tronche CedexFrance; **4**Sabah Wildlife Department, Wisma MuisKota Kinabalu, SabahMalaysia; **5**Biodiversity and Ecological Processes Group, Cardiff School of BiosciencesCardiff University, Cathays Park, CardiffUnited Kingdom; **6**World Wildlife Fund-Malaysia, Kota KinabaluSabahMalaysia; **7**Pittsburgh Zoo, PittsburghPennsylvaniaUnited States of America; Institute of Zoology, Zoological Society of LondonUnited Kingdom

## Abstract

Great apes are threatened with extinction, but precise information about the distribution and size of most populations is currently lacking. We conducted orangutan nest counts in the Malaysian state of Sabah (North Borneo), using a combination of ground and helicopter surveys, and provided a way to estimate the current distribution and size of the populations living throughout the entire state. We show that the number of nests detected during aerial surveys is directly related to the estimated true animal density and that a helicopter is an efficient tool to provide robust estimates of orangutan numbers. Our results reveal that with a total estimated population size of about 11,000 individuals, Sabah is one of the main strongholds for orangutans in North Borneo. More than 60% of orangutans living in the state occur outside protected areas, in production forests that have been through several rounds of logging extraction and are still exploited for timber. The role of exploited forests clearly merits further investigation for orangutan conservation in Sabah.

## Abstract 2

A new aerial method for surveying orangutan densities provides robust estimates of their number and could be used for large- scale surveying of great ape populations in Asia and Africa

## Introduction

The two orangutan species, Pongo abelii in Sumatra and Pongo pygmaeus in Borneo, are threatened with extinction in the near future [1,2]. A prerequisite for conserving great apes in their natural habitat is good knowledge of population distribution, density, and size. However, precise information is still lacking for many orangutan populations living in Borneo, hindering the design of wise strategies for their long-term conservation [3]. Densities of orangutans and other great apes are usually estimated from nest censuses along ground line transects [4,5]. In order to obtain final estimates of great ape population sizes, these densities are extrapolated to large forest blocks identified from maps as being “suitable habitat” for apes. In most surveys, however, the size of the area actually sampled is very small, and the estimates may not be representative of the population status and/or the variety of habitats and human disturbances (such as logging or mining) existing in the entire range of the population [6]. In addition, recent land-use changes (such as poaching), and ecological catastrophes (such as those caused by El Niño) or disease outbreaks do not appear in published maps [1].

The latest estimates available for orangutan populations in the Malaysian state of Sabah (North Borneo) range from 20,000 [7] to less than 2,000 orangutans [1]. Recent land transformation renders these estimates out-of-date [8], and in order to gain precise, up-to-date information, we developed an aerial methodology to assess the entire range of the species in the state precisely. Although some preliminary work using orangutan nest counting from a helicopter was conducted in the past in Sabah and Sarawak [9], this is the first time that aerial surveys have been used to determine population estimates for a great ape species at a state level. This methodology is likely to be useful for documenting the status of great ape populations living in fragmented and exploited forests in Asia and possibly in some parts of Africa.

## Results

### Correlation between Ground and Aerial Nest Densities

Ground densities estimated with Distance 3.5 and aerial densities predicted with our model are given in Table 1. Ground and aerial densities showed a positive correlation with data recorded by the first observer (*R^2^* = 0.86, *n* = 13, *p* < 0.001), by the second observer (*R^2^* = 0.70, *n* = 13, *p* < 0.001), by both observers (*R^2^* = 0.58, *n* = 26, *p* < 0.001), and with the average value obtained for both observers at each site (*R^2^* = 0.83, *n* = 13, p < 0.001).

**Table 1:**
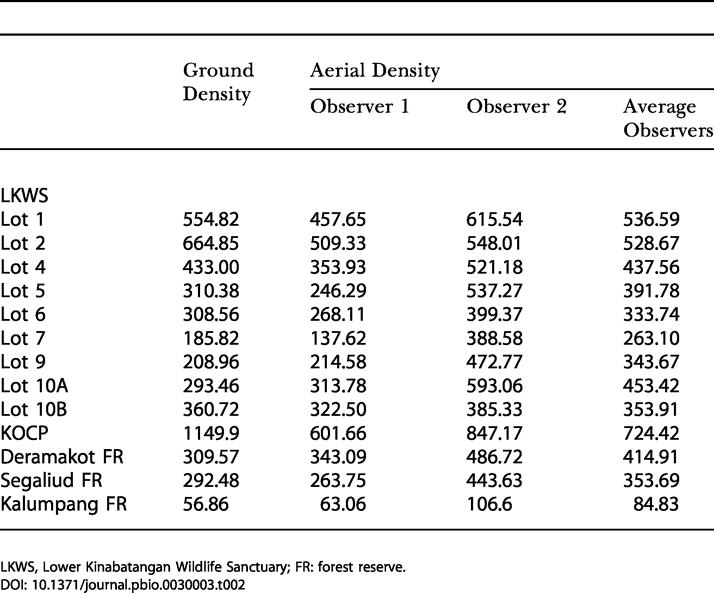
Estimated Ground and Aerial Orangutan Nest Densities (Number of Nests/km^2^) at 13 Different Sites Surveyed during the Orangutan General Census of Sabah

LKWS, Lower Kinabatangan Wildlife Sanctuary; FR: forest reserve

### Orangutan Distribution in Sabah

We recorded 2,708 orangutan nests during ground surveys (225 km of line transects and 300 km of recce walks) and 6,936 nests from the helicopter (1,963 km of aerial lines). The size of the sampling areas ranged from 0.001% to 1% (ground survey) and from 1.8% to 16.9% (helicopter survey, assuming an average strip width of 300 m) of the total size of each forest surveyed (Table 2).

**Table 2:**
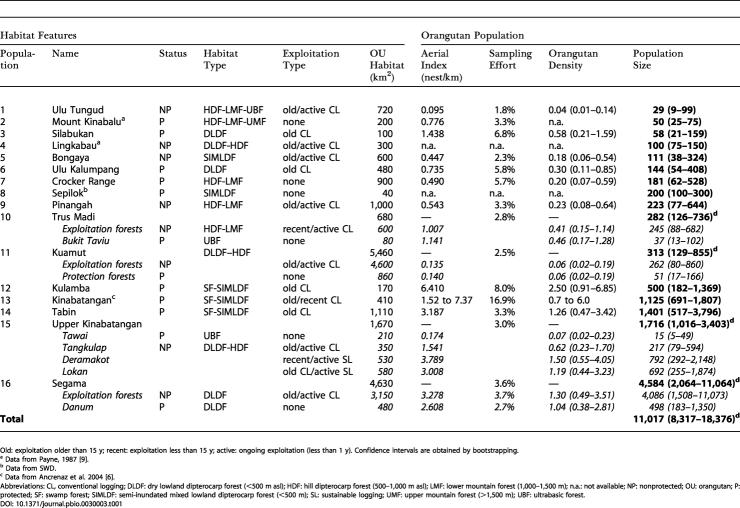
Area Name and Size of Habitat Occupied by Orangutans, Aerial Indexes, Nest and Orangutan Densities, and Final Population-Size Estimates for the 16 Major Orangutan Populations Identified during the Surveys in Sabah, Malaysia, Borneo

Old: exploitation older than 15 y; recent: exploitation less than 15 y; active: ongoing exploitation (less than 1 y)

^a^Data from Payne, 1987 [9]

^b^Data from SWD

^c^Data from Ancrenaz et al. 2004 [6]

^d^Confidence intervals are obtained by bootstrapping

CL, conventional logging; DLDF: dry lowland dipterocarp forest (<500 m asl); HDF: hill dipterocarp forest (500–1,000 m asl); LMF: lower mountain forest (1,000–1,500 m); n.a.: not available; NP: nonprotected; OU: orangutan; P: protected; SF: swamp forest; SIMLDF: semi-inundated mixed lowland dipterocarp forest (<500 m); SL: sustainable logging; UMF: upper mountain forest (>1,500 m); UBF: ultrabasic forest

DOI: 10.1371/journal.pbio.0030003.t002

**Figure 1:**
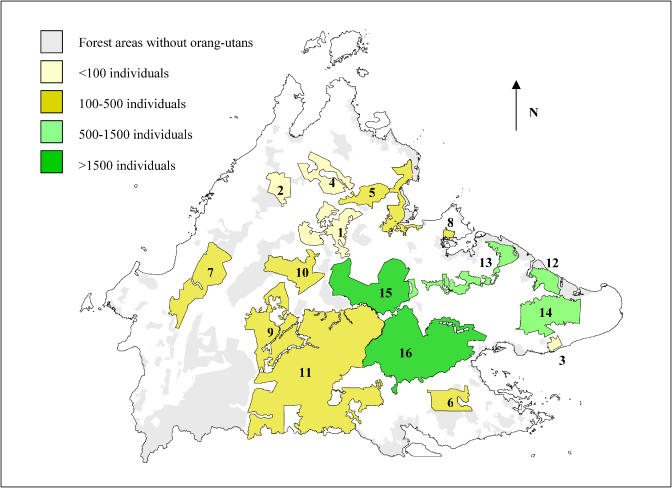
Distribution and Size of the 16 Major Orangutan Populations Identified during the Surveys in Sabah, Malaysia, Borneo

Our surveys confirmed that orangutans were patchily distributed throughout their range in Sabah [7], occurring mainly in the eastern and central parts of the state (Figure 1). Only two significant small and isolated populations were found in the western and northern parts of the state, in Crocker Range National Park (population 7) and Mount Kinabalu National Park (population 1; see Table 2 and Figure 1).

The highest nest abundances were recorded in lowland forests below 300 m asl, although we recorded a few nests as high as 1,300 m asl, which appeared to be the upper altitudinal limit for the species in Sabah. The highest orangutan densities (more than six individuals/km^2^ locally) were identified in the semi-inundated lowland forests of Kinabatangan (population 13) and Segama floodplains (population 16), Kulamba (population 12), and Tabin (population 14). Most of these forests were highly disturbed, fragmented, and located at the edge of newly established oil palm plantations.

Extensive areas of dry lowland dipterocarp forests found in the commercial forest reserves located in the central parts of Sabah (populations 11, 15, and 16) yielded higher orangutan densities in old exploited areas and in areas that were exploited under sustainable logging practices (1.2–2.7 individuals/km^2^, *n* = 4) than in areas where more conventional practices were implemented (0.1–2.0 individuals/km^2^, *n* = 11): Mann–Whitney *U* test, *U* = 5.5, *p* = 0.03. Hunting pressure was low in all these forests ([7]; Kinabatangan Orangutan Conservation Project [KOCP], unpublished data).

### Orangutan Numbers in Sabah

Our surveys showed that about 11,000 orangutans (95% confidence interval: 8,000 to 18,000) were present in Sabah at the time of our surveys (Table 2). Two major orangutan populations were found in logged commercial forest reserves: the Segama forests (population 16, included within the Sabah Foundation forest concession) with about 4,500 individuals, and on the north side of the upper Kinabatangan River (population 15) with about 1,700 individuals (see Table 2 and Figure 1). Four significant populations occurred in isolated protected areas: Tabin Wildlife Reserve (population 14; about 1,400 individuals), Kinabatangan Wildlife Sanctuary (population 13; 1,100 individuals), Kulamba Wildlife Reserve (population 12; 500 individuals), Danum Valley Conservation Area (part of population 16; 500 individuals). The remaining populations were of smaller size, scattered, and isolated.

## Discussion

Aerial surveys are widely used for estimating animal abundance and population trends in open and semi-open landscapes [22]. In Sabah, we report that helicopters can also be used for a forest-dwelling species for (1) directly assessing orangutan distribution, and (2) estimating orangutan population size if aerial surveys are conducted in conjunction with a precalibrating stage based on ground-nest surveys. Aerial nest counts increase the size of the sampling areas significantly, provide a way to survey remote areas that are not accessible from the ground, are faster, and require a lower human investment than classical ground censuses.

Nest detectability from the helicopter depends on observers and canopy structure. Ideally, specific models for deriving nest densities from aerial indexes (number of nests detected per kilometer of flight) should be designed for different human observation skills and for different habitat types. However, observer bias can be avoided if the same team of skilled people conducts the entire survey. The second source of bias could be overcome with the design of several habitat-specific models. Before these types of models are designed, ground-truthing must be conducted in different habitat types in order to validate a baseline model and to determine habitat-specific correction factors when necessary.

Nest parameters used for obtaining the final orangutan density estimates (nest decay rate, daily rate of nest construction) are a major source of inaccuracy in aerial and ground nest surveys [23], and there is a need to investigate interpopulation differences in nest life-span estimates further to produce more precise estimates of orangutan densities [18].

Our survey shows that there are currently about 11,000 orangutans present in Sabah, making the state the main stronghold for the *P. p. morio* subspecies [24]. However, this represents a minimum 35% decline over the past 20 years [7]. This decline is mainly due to habitat loss resulting from the recent conversion of extensive tracts of lowland forests to agriculture [1,8].

The current network of protected areas in Sabah harbors about 4,000 orangutans, representing about 40% of the total number found in the state. About 60% of the total number of orangutans survives in commercial forest reserves subjected to timber extraction, and these forests harbor the largest unfragmented population of the subspecies *P. p. morio* found in Borneo (population 16).

The impacts of forest exploitation on ape abundance and ecology depend on several factors, such as (1) the forest types that existed initially and the quality of the regrowth forest [25], (2) type of habitat exploitation [26,27], (3) hunting pressure [28], and (4) species ecology [29].

Our results tend to indicate that the mosaic of habitats found in the semi-inundated mixed dipterocarp forests that were originally occurring in the floodplains of east Sabah could potentially still harbor a significant number of animals following high disturbance levels (populations 12, 13, and 14). However, we can assume that the very high orangutan densities documented in some of the areas located close to oil palm plantations partly result from the influx of newcomers following recent land conversion to agriculture [7,30]. We can also assume that the response of the forests to logging will directly impact the susceptibility of orangutans to habitat exploitation [7].

Less diverse habitats (dry lowland dipterocarp forests) located in the interior of the state appear to maintain fewer orangutans, particularly following conventional, nonsustainable logging practices (populations 15 and 16). In the extensive tracts of dry lowland dipterocarp forests exploited for timber (populations 15 and 16), our data suggest that, when hunting pressure is low, orangutan abundance is directly related to the degree of logging and associated damage. For these two populations, the highest orangutan densities were identified in Deramakot, a commercial forest reserve (part of population 15) implementing sustainable logging practices [10], suggesting that more conventional, uncontrolled logging activities have a negative impact on orangutan abundance.

Possible inter- and intraspecific differences in general ecology and feeding behavior of orangutans may also influence population responses to habitat disturbances [31], and the results documented in Sabah for *P.p. morio* are not necessarily valid for other Bornean orangutan subspecies and for the Sumatran species [32,33,34,35].

All great ape species require large forest areas to survive. An ecological network combining protected areas with seminatural landscape elements and production forests could be seen as an option to conserve biodiversity, while also providing opportunities for the sustainable use of natural resources [36,37]. However, there is a need for in-depth field studies investigating further the impacts of logging and associated human activities (such as illegal killing) on great ape ecology and survival in order to assess the role of nonprotected forests for ape conservation.

Finally, aerial nest surveys may also be of use in Africa, although it may be difficult to detect nests of African great apes from a helicopter because they tend to be lower in the canopy and it may be impossible to distinguish between gorilla and chimpanzee nests in those areas where the two species are sympatric [4].

## Materials and Methods

### 

#### Study area: Sabah

**Figure 2:**
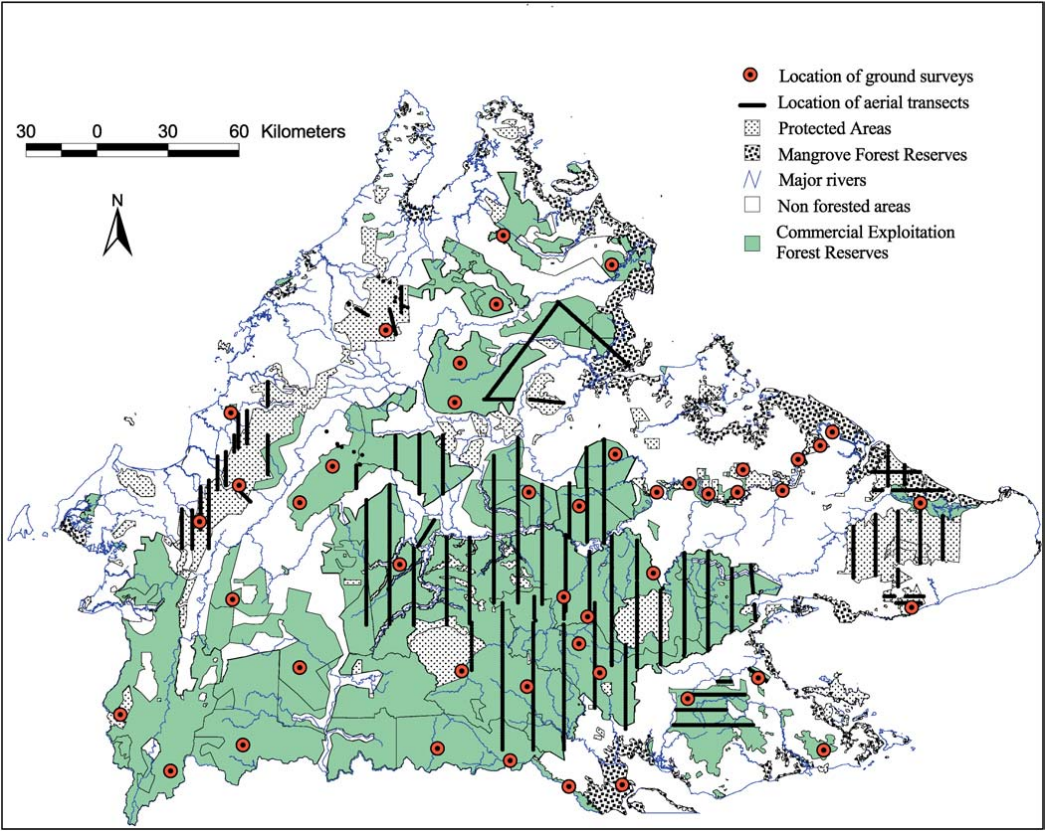
Location of Ground and Aerial Surveys during the Orangutan Census in Sabah, Malaysia, Borneo Aerial transects are not shown for the Lower Kinabatangan forests.

Sabah covers about 76,000 km^2^ in the northern portion of the island of Borneo. It is one of the 13 states in the federation of Malaysia. Approximately half of the total land mass is covered with forests (Figure 2). Commercial forest reserves are designated for timber extraction and represent 76% of all forests in Sabah [8]. Sustainable logging practices (proper forest management plan and precise extraction planning, selective and reduced-impact logging) are currently implemented in Deramakot Forest Reserve (part of population 15) and are in the process of being generalized to other commercial forest reserves where more conventional practices were still implemented in the recent past [10]. The remnant forests have various protection statuses, but most of them have been logged using conventional forestry practices at least once in the past (Table 2).

#### Helicopter census.

All major forest blocks in Sabah were identified from 1:50,000 vegetation maps, and these were divided into 16 different blocks. For each block, we determined a systematic stratified sampling using equidistant parallel line transects, the location of the first line being randomly selected (Figure 2). Because the specific topographical features (steep slopes and high altitudes) encountered over Crocker Range and Kinabalu National Parks prevented the helicopter from following a random pattern of transects, the location of our aerial lines followed valleys in these two blocks. Aerial censuses were carried out with a small-type Bell 206 Jet ranger helicopter. Helicopter speed and height were kept constant at about 70 km/h and 60–80 m above the forest canopy. The copilot recorded the precise flight path location with a Global Positioning System every 30 s and monitored altitude, forest type (semi-inundated vs dry), signs of human activities, and forest disturbance continuously. Four types of disturbances were distinguished during aerial surveys: (1) no disturbance: tall and large trees; rather closed canopy; no sign of human exploitation; (2) old exploited forests (timber extraction was conducted more than 15 y prior to the survey): logging roads and stamping areas colonized by pioneer tree species such as *Macaranga* sp. (crown shape and color easily distinguishable from other tree species), some emergent trees; (3) recently exploited forests (timber extraction was conducted less than 15 y ago): logging roads not entirely colonized by pioneer tree species; few emergent trees; (4): active exploitation: logging activities were ongoing at the time of the survey.

From the back seats, two observers looked for orangutan nests from either side of the helicopter. All visible nests were recorded. It was impossible to estimate the impact of nest age on nest detection, and the observers acknowledged that a few days-old fresh nests (still green in color) and nests at their latest stages of decay (just a few branches visible) were difficult to detect in the canopy. These nest categories are likely to have been underdetected. The two observers indicated all sightings to a nest recorder seated between them. The nest recorder noted the number of nests detected by the observers per each 30-s period. All crew members were in constant radio contact during the flights. After the flight, data collected by the copilot and the nest recorder were matched in order to determine the location of all sightings along the aerial line transect precisely. The same team of observers conducted all aerial surveys in order to avoid the observer bias.

For technical reasons, it was impossible to fit external devices to the helicopter to estimate the distance of the nests to the aerial transects. This prevented us from determining the detection function from our data alone [11]. Trailing tapes placed on the aircraft window limited the observers' field of view to a strip of approximately 150 m wide on either side of the aircraft. However, fluctuations in canopy's height prevented the direct determination of the exact width of the sampling area.

#### Ground censuses.

Because the proportion of the actual nest population existing in the forest that was detected from the helicopter was unknown, it was impossible to directly estimate nest densities from our aerial results [9]. We thus designed a calibration function relating nest density estimated from the ground to the number of nests detected per kilometer of flight (aerial nest index) by comparing the aerial results with results from extensive ground surveys carried out in 13 patches of old and recently disturbed forests located in the Kinabatangan Wildlife Sanctuary [6], and Deramakot, Kalumpang, and Segaliud forest reserves.

Nest densities and their variances were estimated by ground line transects using distance sampling [11,12]. A set of line transects was randomly selected and the perpendicular distance of each nest to the transect was carefully recorded [6]. Densities were computed using the software Distance 3.5 [13]. For each transect, the truncation level was set following identification of outliers from box plots (outliers being values higher than 1.5 box-lengths from the 75^th^ percentile). Heaping was assessed from histograms, and data were grouped where necessary [11,14]. The probability of nest detection was estimated with models combining density functions (uniform, half-normal, and hazard-rate) with adjustments (cosine, simple, Hermite polynomials). The model with the lowest Akaike's Information Criterion was selected for each site [15]. The adequacy of the selected model to the perpendicular distances was assessed by a chi-square goodness-of-fit test on grouped data [11]. Finally, we estimated the variance of nest density using nonparametric bootstrapping to handle sources of variation, such as model selection uncertainty [11]. Results are given in Table 1, and are extensively described in [6].

#### Estimation of nest density from aerial indexes.

The calibration function relating absolute nest density to aerial nest index stipulated that the logarithm of the orangutan nest density *D^* was a linear function of both the logarithm of the aerial index *AI* and the observer effect *obs*, plus their interaction, in order to include possible differences between observers. We weighted the general regression by the estimated variances of ground nest densities, thus giving a greater emphasis to precise density estimates. The least squares method was used for model fitting by incorporating weights 1/σ^_log(D^_*i*_)_ where σ^_log(D^_*i*_)_ was the estimated standard error of the estimated nest density logarithm in forest area *i,* given by



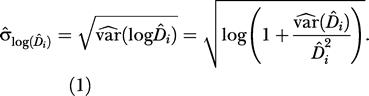



Then, assuming that the densities were log-normally distributed, the overall regression model was conveniently written with a matrix notation as



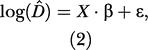



where *D^* was a 26 × 1 vector of the orangutan nest densities (13 points per observers), and *X* was the matrix of covariates:



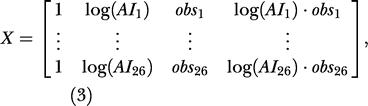



β was a 4 × 1 vector of parameters to be estimated, and ɛ was a 26 × 1 vector of errors with multivariate normal distribution *N*_26_(0,Σ) , where Σ was a 26 × 26 matrix with σ^2^ · *diag*(var(logD^_*i*_)) in the diagonal and zeroes elsewhere.

To simplify, (2) was *D^* rewritten using the quantities log (*D^*)*_w_* = *W* · log(*D^*), *X_w_* = *W* · *X*, and ɛ*_w_* = *W* · ɛ, with *W* = *diag*(σ^^−1^_log(D^_i_)_ as



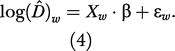



Unlike ɛ, ɛ*_w_* has a more familiar distribution *N*_26_(0, σ^2^·*I*_26_), allowing the use of linear regression tools to estimate model parameters via least square theory. We used the backward model selection procedure [15] to select between models. The first regression model to be tested included all covariates. Covariates with the highest *p* value and greater than a 10% cutoff were then removed one by one, and each new model was retested until all *p* values of the remaining covariates were less than the cutoff value. We assessed the goodness of fit of the best model by computing the coefficient of determination *R^2^*. The best model supported by the data considered only the aerial index effect (*R^2^* = 0.9587, *F* ^23^_2,0.05_ = 3.42 , *p* < 0.001, on the logarithmic scale) with



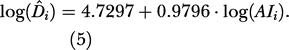



**Figure 3:**
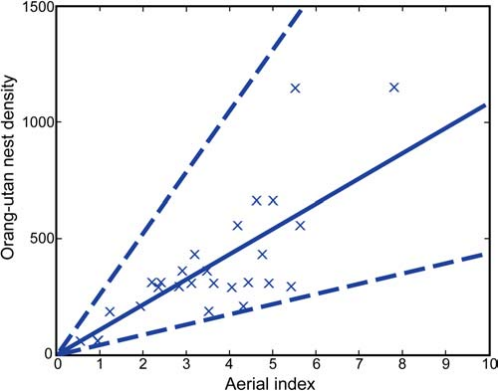
Graph Showing the Predicted Orangutan Nest Density as a Function of Aerial Indexes The plain line is the fitted line via model (5), and dashed lines are prediction intervals; *n* = 13 sites, 2 observers.



Using this model (5), we predicted an orangutan nest density from any new aerial index values, *AI_0_,* recorded during helicopter flights, as



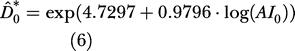



(Figure 3). This model was applied to all the forests that were surveyed only by helicopter. A 95% confidence interval for the predicted orangutan nest density was built up on the logarithmic scale as

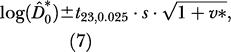



with



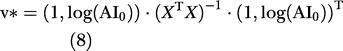



and *X*, the matrix defined above, once the observer effect had been removed, *s* ^2^ the residual mean square up to a constant that was an estimate of σ^2^, and *t*_23,0.025_ the appropriate two-sided *t*-distribution percentile [16]. Following [11], this interval was then back-transformed to obtain a final confidence interval for the predicted orangutan nest density as



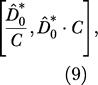



where



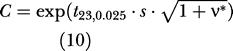



and



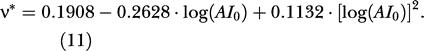



A numerical application gave



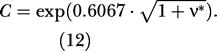



These intervals are shown in Figure 3.

#### Correction factors and habitat types.

Model (5) was obtained with results from the old and recently exploited semi-inundated mixed lowland dipterocarp forests of Kinabatangan. We tested its validity by comparing nest densities predicted from aerial data and estimated from ground line transects at several sites. We found no significant differences for five sites of old and recently exploited dry lowland dipterocarp forests (*t*-test, *n* = 5, *t* = −1.738, *p* = 0.157; 95% confidence interval of the difference: −110 to 25; ratio between ground and aerial nest densities = 0.94). This result showed that nest detectability was similar in forests that had been exploited for timber in Sabah and in Kinabatangan. Thus, we used the baseline model without any correction for all recent and old exploited forests of the state. Exploited swamp forests had a very open canopy, and predicted aerial nest densities were higher than estimated ground densities, although the difference was not significant (*t*-test, *n* = 3, *t* = −3.331, *p* = 0.08; 95% confidence interval of the difference: −384 to 49; ratio = 0.54). However, in order to not overestimate the final densities, we applied a correction factor of 0.54 to aerial indexes obtained in two areas of extensive exploited swamp forests (parts of populations 12 and 14). In primary lowland dipterocarp forest, the predicted aerial nest density was lower than the estimated ground density in the only site that was tested (*n* = 1; 392 nests/km^2^ vs 592 nests/km^2^; ratio = 1.5). Aerial indexes obtained for Danum (part of population 16), the only site of primary lowland forest with a significant orangutan population assessed during our survey, were multiplied by a correction factor of 1.5.

#### Estimation of orangutan density from nest density.

The actual orangutan density D^_ou_ was estimated using



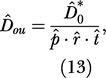



with D^^*^_O_ the predicted nest density, p^ the estimated proportion of nest builders, t^ the estimated nest decay rate, and r^ the estimated daily rate of nest production [4].

The proportion of nest builders has been estimated as 0.9 for orangutans [5,17,18]. The daily rate of nest production is currently available for only two Bornean orangutan populations: 1.005 in Kinabatangan [18] and 1.163 in Gunung Palung [17]. In order to take into account interpopulation variability in orangutan nesting behavior and to obtain more conservative estimates of orangutan densities in Sabah, we used an average value of 1.084 for our survey (with an associated coefficient of variation of 0.063). Nest decay rate varies with forest type [5,18], and the most reliable estimates are obtained via direct monitoring of the survival of a sufficient number of nests [19]. Such estimates for t^ are available for only two sites in Borneo: Gunung Palung, with 399 d and 258 d in mixed semi-inundated lowland and dry lowland forests, respectively [17]; and Kinabatangan with 202 d [18]. Since specific nest decay rates were not available for the different forests surveyed in Sabah, we considered an average t^ value of 286.3 d (coefficient of variation: 0.373). Using the δ-method [20], a 95% confidence interval for the estimated D^_ou_ was built up as



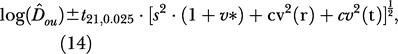



with cv as the coefficient of variation and the other quantities as already defined (see above). This interval was then back-transformed to obtain a confidence interval for D^_ou_ as



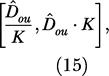



where



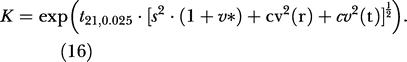



The numerical application gave



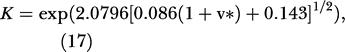



with



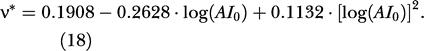



#### Estimation of orangutan population size.

The results of the ground and aerial surveys were processed with a geographic information system (Arcview 4.1; ESRI, Redlands, California, United States), using a combination of administrative maps and satellite images. When necessary, we stratified each forest block according to (1) disturbance type: no disturbance, old or recently exploited forests, ongoing exploitation; (2) altitude: lowland, below 500 m above sea level (asl); upper land, 500–1,000 m asl; lower mountain, 1,000–1,500 m asl; mountain, above 1,500 m asl; and (3) habitat type: swamp forests, semi-inundated mixed lowland dipterocarp forests, dry lowland dipterocarp forests. We then determined the percentage of habitat actually occupied by orangutans as the ratio between the total length of aerial transects and the length flown over areas with no visible orangutan nests (large areas with no trees, such as grasslands, large forest gaps, rivers, and oxbow lakes). This percentage was applied to the total size of each forest block determined from maps in order to estimate the final size of “habitat occupied by orangutans.” We then multiplied the estimated orangutan densities by the estimated size of orangutan habitat occupation to obtain overall population estimates.

A confidence interval for the population estimate was computed via parametric bootstrapping for the whole population in Sabah [21]. We assumed a normal distribution for population sizes extracted from the literature, and we assumed a log-normal distribution for other populations, with parameters given by formula (4). Values were sampled from their appropriate distribution and summed to obtain the whole population size. We repeated these two steps 1,000 times to obtain the bounds of a 95% bootstrapped confidence interval as the 25th and 975th largest values. We adopted a similar procedure for the subpopulations constituting populations 9, 11, 15, and 16.

#### Survey efforts.

Over a 2-y period (2002–2003), ground surveys (using a combination of line transects and recce walks for a total effort of 1,100 “man days” of fieldwork) and aerial surveys (72 “man days”) were conducted in all major forests of the state (Figure 2). Recce walks were conducted to assess the presence/absence status of orangutans in areas with harsh topographical features or with extremely low orangutan abundance. Results from recce walks were not used to estimate nest densities.

## Abbreviations

asl: above sea level
KOCP: Kinabatangan Orang-utan Conservation Project
